# The short Thai version of functional outcomes of sleep questionnaire (FOSQ-10T): reliability and validity in patients with sleep-disordered breathing

**DOI:** 10.1007/s11325-024-03024-1

**Published:** 2024-05-15

**Authors:** Kawisara Chaiyaporntanarat, Wish Banhiran, Phawin Keskool, Sarin Rungmanee, Chawanont Pimolsri, Wattanachai Chotinaiwattarakul, Auamporn Kodchalai

**Affiliations:** 1 Department of Otorhinolaryngology, Faculty of Medicine Siriraj Hospital, Mahidol University, Bangkok, Thailand; 2 Siriraj Sleep Center, Faculty of Medicine Siriraj Hospital, Mahidol University, Bangkok, Thailand; 3 Neurology Division, Department of Medicine, Faculty of Medicine Siriraj Hospital, Mahidol University, Bangkok, Thailand; 4 American Board of Sleep Medicine, Department of Otorhinolaryngology, Faculty of Medicine Siriraj Hospital, Certified International Sleep Specialist, Mahidol University, 2 Wanglang Road, Bangkok Noi, Bangkok, 10700 Thailand

**Keywords:** Functional Outcome of Sleep Questionnaire, FOSQ-10, Thai, Obstructive sleep apnea, OSA, Sleep-disordered breathing, SDB

## Abstract

**Purpose:**

The study is to evaluate reliability and validity of the short Thai version of Functional Outcome of Sleep Questionnaire (FOSQ-10T), in patients with sleep disordered breathing (SDB).

**Methods:**

Inclusion criteria were Thai patients with SDB age ≥ 18 years old who had polysomnography results available. Exclusion criteria were patients unable to complete questionnaire for any reason, patients with a history of continuous antidepressant or alcohol use, and underlying disorders including unstable cardiovascular, pulmonary, or neurological conditions. All participants were asked to complete the FOSQ-10 T and Epworth sleepiness scales (ESS). Of these, 38 patients were required to retake FOSQ-10 T at 2–4 weeks later to assess test–retest reliability, and 19 OSA patients treated with CPAP were asked to do so at 4 weeks following therapy to assess questionnaire’s responsiveness to treatment.

**Results:**

There were 42 participants (24 men, 18 women), with a mean age of 48.3 years. The internal consistency of the FOSQ-10T was good, as indicated by Cronbach’s alpha coefficient of 0.85. The test–retest reliability was good, as indicated by intraclass correlation coefficient of 0.77. The correlation between the FOSQ-10T and ESS scores (concurrent validity) was moderate (*r* =  − 0.41). The scores of FOSQ-10T significantly increased after receiving adequate CPAP therapy, showing an excellent responsiveness to treatment. However, there was no significant association between FOSQ-10T scores and OSA severity measured by apnea–hypopnea index.

**Conclusions:**

The FOSQ-10T has good reliability and validity to use as a tool to assess QOL in Thai patients with SDB. It is convenient and potentially useful in both clinical and research settings.

**Supplementary Information:**

The online version contains supplementary material available at 10.1007/s11325-024-03024-1.

## Introduction

The term “sleep-disordered breathing” (SDB) refers to a category of high prevalent sleep disorders that are distinguished by abnormal breathing patterns when the patient is asleep. Its negative consequences, especially obstructive sleep apnea (OSA), include excessive daytime sleepiness, high blood pressure, poor quality of life (QOL), cardiometabolic diseases, and sensorineural hearing loss [1–6]. In addition to lowering these possible morbidities and deaths, improving the patients’ QOL is a key goal of appropriately treating SDB [7].

Currently available instruments to assess health-related QOL in individuals with sleep disorders include general and disease-specific questionnaires [5, 8, 9]. The Functional Outcomes of Sleep Questionnaire (FOSQ-30), however, is perhaps one of the most widely utilized [10]. The questionnaire is a standardized self-report form consisting of 30 items that cover various domains including sexual relationships, general productivity, activity level, vigilance, and social consequence. Each of the FOSQ-30 items is given a score between 0 and 4, with a higher score representing a higher quality of life. However, one of the FOSQ-30’s drawbacks is the somewhat lengthy time required to respond to all questions (a total of 20–25 min). The original authors subsequently developed a shortened version (FOSQ-10) to make the QOL assessment easier and more efficient while still maintaining all crucial components [11]. Unfortunately, there is currently no validated version of this tool available for Thai patients.

The FOSQ-10 has been used to study the effects of therapeutic interventions such as functional septorhinoplasty [12], continuous positive airway pressure (CPAP) treatment [13, 14], oral appliance [15, 16], and the effects of gastroesophageal reflux disease [17]. Previous research also showed that the FOSQ-10 was validated across a number of languages and ethnics [18–20]. In Iranians, a study found that the FOSQ-10 was comparable in meaning to the original version [18]. In Peruvians, the Spanish version of FOSQ-10 showed good internal consistency, construct validity, and sensitivity to change in patients with OSA who received treatment [19]. In Chinese, a study reported that the FOSQ-10 was a valid and reliable instrument for identifying the effects of sleep-related impairment in women during pregnancy [20]. Yet, no study has looked into its use in Thai people.

The primary objective of this study was to evaluate reliability and validity of the short Thai version of Functional Outcomes of Sleep Questionnaire (FOSQ-10T). The secondary objectives were to evaluate (1) QOL of OSA patients pre- and post-CPAP treatment, (2) QOL of SDB patients across different AHI severity, and (3) the correlation between scores of the FOSQ-10T and Epworth sleepiness scale (ESS).

## Material and methods

This observational, prospective research was approved by the Siriraj Institutional Review (SIRB), COA Si 258/2021. The study was conducted between November 2021 and February 2022. All participants gave their informed consent.

### Subjects and allocation

The inclusion criteria were Thai patients with SDB who were at least 18 years old and had polysomnography (PSG) results available. The exclusion criteria were patients who were unable to complete questionnaire for any reason, those with a history of long-term sedatives, antidepressants, or alcohol use, and those with underlying medical conditions that would significantly impair QOL.

**Fig. 1:**
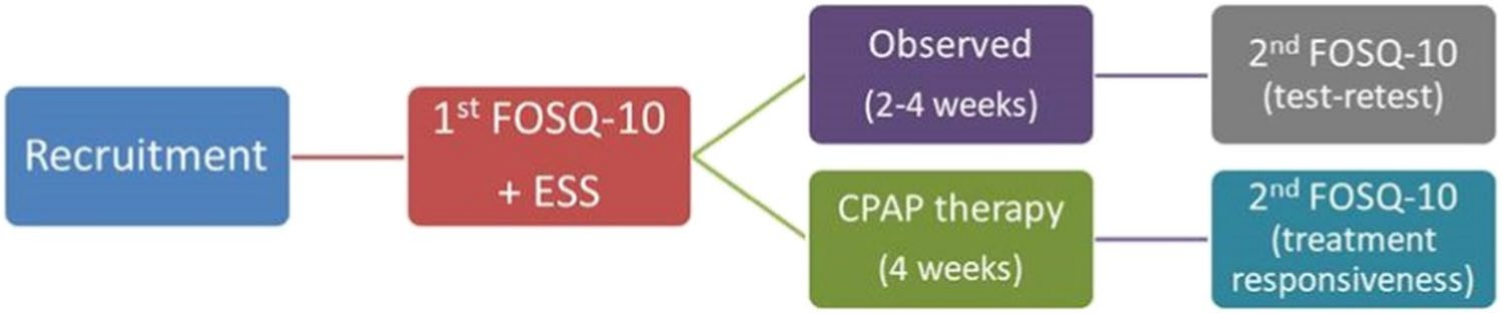
The flow chart of the study; FOSQ-10, the short form of the Functional Outcomes of Sleep Questionnaire; ESS, Epworth Sleepiness scale; CPAP, continuous positive airway pressure

All participants were asked to complete the FOSQ-10T and ESS. Of these, 38 patients were asked to retake the FOSQ-10T between two and four weeks later in order to evaluate test–retest reliability, and 19 patients with OSA who were receiving CPAP were asked to retake the questionnaire 4 weeks later in order to evaluate the questionnaire’s responsiveness to treatment, as presented in the flow chart (Fig. 1).

### The short Thai version of Functional Outcomes of Sleep Questionnaire (FOSQ-10T)

The FOSQ-10T is a 10-item self-reported questionnaire that measures the impact of sleep disturbances on daily functioning. Though it is a condensed form of the FOSQ-30, it still includes all of the important domains: activity level (three items), vigilance (three items), sexual relationships (one item), general productivity (two items), and social outcome (one item). Every item is assigned a number between 0 and 4, where a higher number corresponds to a higher QOL. With permission of Professor Terri Weaver, the original developers [10] graciously provided us with the FOSQ-10T (see Supplementary information the appendix) for use in this study. Standard processes were used to translate it both forward and backward from English to Thai.

### Epworth sleepiness scales (ESS)

The ESS is a self-administered questionnaire used to assess an individual's subjective level of sleepiness. It comprises of eight questions that ask the respondent to rate their likelihood of dozing off or falling asleep in a range of everyday scenarios, such as sitting and conversing with someone, watching television, or riding as a passenger in a car. The scores range from 0 to 3, where a higher number indicates greater sleepiness. In this study, we use a validated Thai version of ESS with permission [21] (see Supplementary information the appendix).

### Statistical analysis

The categorical data were presented as numbers and percentages, whereas the continuous data were presented as mean ± standard deviation (SD). The Cronbach’s alpha coefficient was used to evaluate the internal consistency for reliability and the intraclass coefficient (ICC) was used to evaluate test–retest reliability. The discriminant validity of the FOSQ-10T among each SDB severity was evaluated using a Kruskal–Wallis one-way analysis of variance (ANOVA), and the concurrent validity of the FOSQ-10T and ESS was evaluated using a scatter plot and a Pearson correlation coefficient. A significance level of *p* < 0.05 was employed to denote statistical significance. The Statistical Package for the Social Science (SPSS) version 22, International Business Machines Corporation, Armonk, NY, USA, was utilized to conduct the statistical analyses.

## Results

For this study, 42 participants (24 men and 18 women) with a mean age of 48.3 ± 15.3 years and a mean BMI of 27.9 ± 5.4 kg/m^2^ were recruited. The apnea–hypopnea index (AHI) and mean ESS scores for the group were 38.6 ± 29.8 events/h and 7 ± 3.8, respectively. Among the participants, 16 (38.1%) had hypertension, 11 (26.2%) had dyslipidemia, and 7 (16.7%) had underlying diabetes mellitus.

### Reliability

Cronbach’s alpha coefficient of the FOSQ-10T ranged from 0.82 to 0.85 for all 10 items, and the removal of any items did not significantly alter the result (Table 1). This suggested a high level of internal consistency. With an ICC of 0.77 and a 95% confidence interval (CI) of 0.60–0.88, the FOSQ-10T demonstrated good test–retest reliability.

**Table 1:** Cronbach’s alpha coefficient for internal consistency

FOSQ-10 T	Mean item score	Cronbach’s alpha if item deleted
General productivity item1	2.74	0.85
General productivity item2	2.70	0.83
Social outcomes	2.86	0.83
Activity level item1	3.10	0.85
Activity level item2	2.95	0.83
Activity level item3	2.81	0.83
Vigilance item1	2.33	0.83
Vigilance item2	2.02	0.82
Vigilance item3	2.88	0.83
Intimacy and sexual relationships	2.52	0.84

*FOSQ-10T,* the short Thai version of the Functional Outcomes of Sleep Questionnaire

### Validity

The concurrent validity between FOSQ-10T and ESS scores was shown by a scatter plot (Fig. 1) and Pearson correlation coefficient of − 0.41 (*p* = 0.01) which indicated that there was a moderate correlation. However, the results of the discriminant validity analysis using the Kruskal–Wallis one-way ANOVA indicated that there was no significant correlation between the OSA severity as determined by the AHI and the FOSQ-10T scores (Table 2).

**Table 2:** FOSQ-10 T scores in obstructive sleep apnea patients with varying degrees of severity

FOSQ-10 T	Mild OSA	Moderate OSA	Severe OSA	*P* value
General productivity	2.50 ± 0.83	2.60 ± 0.94	3.12 ± 1.12	0.14
Social outcomes	2.22 ± 1.79	3.40 ± 0.70	2.90 ± 1.55	0.38
Activity level	2.70 ± 0.82	3.37 ± 0.25	3.07 ± 0.93	0.07
Vigilance	2.26 ± 1.16	3.13 ± 1.08	2.25 ± 1.27	0.09
Intimacy and sexual relationships	1.89 ± 1.62	2.30 ± 1.70	3.00 ± 1.55	0.14
Total score	**13.02 ± 4.11**	**15.65 ± 3.10**	**15.61 ± 3.30**	**0.83**

Data are presented as mean ± standard deviation

*FOSQ-10T,* the short Thai version of the Functional Outcomes of Sleep Questionnaire, *OSA* obstructive sleep apnea

### Responsiveness

**Fig. 2:**
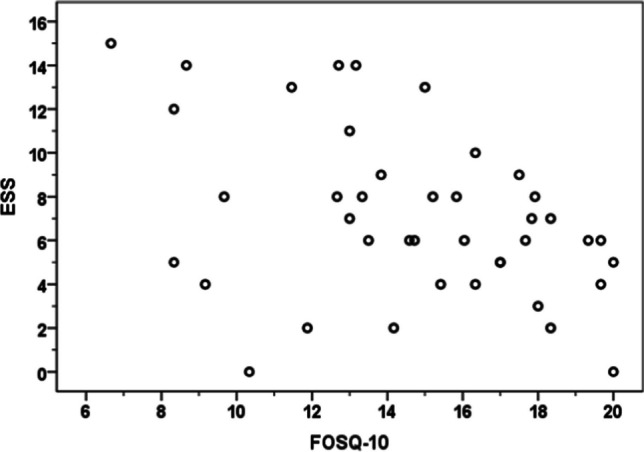
The scatter plot of ESS score and FOSQ-10; FOSQ-10, the short Form of the Functional Outcomes of Sleep Questionnaire; ESS, Epworth sleepiness scale; CPAP, continuous positive airway pressure

The mean FOSQ-10 T scores across all domains and the overall scores considerably improved after receiving adequate CPAP therapy (Table 3). This proved that the questionnaire had an excellent responsiveness to treatment (Fig. 2).

**Table 3:** FOSQ-10 T scores before and after continuous positive airway pressure therapy

FOSQ-10 T scores	Pre-treatment (mean ± SD)	Post-treatment (mean ± SD)	*P* value
General productivity	2.88 ± 1.15	3.60 ± 0.94	0.00 *
Social outcomes	3.20 ± 1.28	3.60 ± 1.23	0.03 *
Activity level	3.13 ± 0.70	3.88 ± 1.96	0.00 *
Vigilance	2.43 ± 1.30	3.00 ± 1.37	0.00 *
Intimacy and sexual relationships	2.85 ± 1.53	3.30 ± 1.45	0.03 *
Total score	**15.53 ± 3.11**	**18.70 ± 1.43**	0.03 *

Data are presented as mean ± standard deviation. *P < 0.05 was used to indicate statistical significance

*FOSQ-10 T* the short Thai version of the Functional Outcomes of Sleep Questionnaire

## Discussion

When treating patients with SDB, the QOL of the patients is an important consideration that cannot be overlooked. While there are other instruments to assess this issue, the FOSQ-30 is likely one of the most widely utilized disease-specific questionnaires. However, its disadvantage is that answering every question takes a substantial amount of time. As a result, the original authors eventually developed a shortened version (FOSQ-10) to streamline and improve the effectiveness of the QOL evaluation while maintaining all crucial elements [11]. Comparable to the original, this updated version has demonstrated good validity and reliability [10]. It has been used to evaluate the effects of various therapeutic interventions [12, 14, 17] and has been validated in several other languages, including Spanish, Persian, and Chinese [18–20].

This study is most likely the first to report on the validity and reliability of the FOSQ-10T in Thai patients with SDB. The results of our study showed that the Cronbach’s alpha coefficient, which measures internal consistency, was 0.85, indicating good reliability. This closely resembles the original version [10] and studies conducted in Chinese, Spanish, and Persian [18–20] that found a Cronbach’s alpha of 0.84–0.87.

The results of the present study showed that the FOSQ-10T has good test–retest reliability with an ICC of 0.77. This could suggest that the tool is reliable when applied repeatedly to the same Thai population. It should be mentioned that the ICC of this study was comparable to that of the Chinese study (ICC of 0.73) and another Thai version of the FOSQ-30 (ICC of 0.70) [5], but it was lower than that of the Iranian study (ICC of 0.92). A straight comparison of ICC values across studies, however, would not always be appropriate because different study designs, populations, and measuring techniques can all have an impact on the values.

According to the study, there was a moderately negative correlation between the FOSQ-10T and the ESS (*r* =  − 0.41). This association is similar to that of the Iranian version [18] and another Thai version of FOSQ-30 [5]. Our finding, however, diverged from that of the Spanish [19] and Chinese studies [20], which did not discover any significant relationships between the FOSQ-10 T and ESS scores.

Among the OSA patients with different AHI severities in this study, there were no statistically significant differences in the FOSQ-10T scores. Mild OSA had the lowest scores, whereas moderate OSA had the highest. These, however, differ from the original English [11], Iranian [18], and Spanish [18, 19] versions which showed moderate degrees of discriminating validity.

Not surprisingly, after therapy, the FOSQ-10T scores of participants in this study who used CPAP appropriately improved significantly. These findings were in line with a number of other studies; thus, it may indicate that the questionnaire had a high degree of treatment responsiveness.

This study may have some limitations. First, because the FOSQ-10T  scores were subjectively evaluated by individuals, bias cannot be avoided. Second, the FOSQ-10T and the original FOSQ-30 were not directly compared, so there is a chance that both of them will produce different results when applied. Furthermore, only relatively healthy SDB patients were assessed in this study. For this reason, our findings could not be directly applied to patients suffering from critical illnesses such as heart failure, stroke, or chronic renal diseases, or to patients with other sleep disorders including insomnia or hypersomnolence due to central origin. It is recommended that further study be done in populations with varying characteristics or manifestations.

## Conclusion

The results of this study indicate that the FOSQ-10T is a valid and reliable tool for evaluating QOL in Thai patients with SDB. In clinical practice, physicians may utilize the questionnaire to monitor therapy results and customize interventions to fit specific patient needs. In research, the FOSQ-10T may be used to evaluate the effectiveness of various therapeutic or diagnostic approaches.

### Supplementary Information

Below is the link to the electronic supplementary material.

Supplementary file1 (PDF 163 KB)

## Data Availability

To comply with general data protection regulation and to protect people’s privacy, the raw data for this dataset is not publicly accessible.
