# Supplementary material for: The short Thai version of functional outcomes of sleep questionnaire (FOSQ-10T): reliability and validity in patients with sleep-disordered breathing
Source: Sleep Breath. 2024 May 15;28(4):1701–6. doi: 10.1007/s11325-024-03024-1 (PMC11303584; doi:10.1007/s11325-024-03024-1)
Supplement: Supplementary file 1 — Supplementary file1 (PDF 163 KB) [file 11325_2024_3024_MOESM1_ESM.pdf]

แบบสอบถามผลลัพธ์การทำหน้าที่ของการนอนหลับ-ฉบับย่อ (Thai version)

(The Functional Outcome of Sleep Questionnaire- short version)

บางคนมีความยากลำบากในการทำกิจกรรมประจำวันเมื่อมีรู้สึกอ่อนล้าหรือง่วงนอน แบบสอบถามฉบับนี้มีวัตถุประสงค์เพื่อประเมินว่าโดยทั่วไปแล้วท่านมีความยากลำบากในการทำกิจกรรมต่างๆ เมื่อมีอาการอ่อนล้าหรือง่วงนอนมาก โดยความง่วงนอนหรืออ่อนล้า ในที่นี้หมายถึง ความรู้สึกที่ท่านไม่สามารถจะทำให้ตาสว่าง หรือมีอาการสัปหงก รู้สึกเหมือนจะเผลอหลับ โดยที่ไม่ใช่เป็นความรู้สึกเหนื่อยหรืออ่อนล้าจากภายหลังการออกกำลังกาย

คำชี้แจง : กรุณาเลือกคำตอบในช่องว่างเพียงคำตอบเดียวที่เป็นจริงสำหรับตัวท่าน

**กิจกรรม**	**ไม่ได้มีกิจกรรมนี้** (0)	**ระดับความยากลำบาก**			
		**ไม่มีความยากลำบาก** (4)	**เล็กน้อย** (3)	**ปานกลาง** (2)	**มาก** (1)
1. ท่านมีความยากลำบากในการมีสมาธิกับสิ่งที่ทำเนื่องมาจากท่านรู้สึกง่วงนอนหรืออ่อนล้า					
2. ท่านมีความยากลำบากในการจดจำสิ่งต่างๆเนื่องมาจาก					

ท่านรู้สึกง่วงนอนหรืออ่อนล้า					
3. ท่านมีความยากลำบากในการขับขี่ยานยนต์เป็นระยะทางใกล้ๆ เนื่องมาจากท่านรู้สึกง่วงนอนหรืออ่อนล้า					
4. ท่านมีความยากลำบากในการขับขี่ยานยนต์เป็นระยะทางไกลๆ เนื่องมาจากท่านรู้สึกง่วงนอนหรืออ่อนล้า					
5. ท่านมีความยากลำบากในการไปเยี่ยมเยือนครอบครัวหรือเพื่อนๆ ของท่าน เนื่องมาจากท่านรู้สึกง่วงนอนหรืออ่อนล้า					
**กิจกรรม**	**ไม่ได้มีกิจกรรมนี้** (0)	**ระดับความยากลำบาก**			
		**ไม่มีความยากลำบาก** (4)	**เล็กน้อย** (3)	**ปานกลาง** (2)	**มาก** (1)
6. ความสัมพันธ์ระหว่างครอบครัว เพื่อน หรือเพื่อนร่วมงานของท่านเปลี่ยนแปลงไปเนื่องมาจากท่านรู้สึกง่วงนอนหรืออ่อนล้า					

7. ท่านมีความยากลำบากในการที่จะดูโทรทัศน์ หรือชมภาพยนตร์เนื่องมาจากท่านรู้สึกง่วงนอนหรืออ่อนล้า					
8. ท่านมีความยากลำบากในการที่จะทำให้ตัวเองรู้สึกกระฉับกระเฉงในตอนเย็นเนื่องมาจากท่านรู้สึกง่วงนอนหรืออ่อนล้า					
9. ท่านมีความยากลำบากในการที่จะทำให้ตัวเองรู้สึกกระฉับกระเฉงในตอนเช้าเนื่องมาจากท่านรู้สึกง่วงนอนหรืออ่อนล้า					
**กิจกรรม**	**ไม่ได้มีเพศสัมพันธ์** (0)	**ไม่เปลี่ยนแปลง** (4)	**เปลี่ยนแปลงเล็กน้อย** (3)	**เปลี่ยนแปลงปานกลาง** (2)	**เปลี่ยนแปลงมาก** (1)
10. ความต้องการการใกล้ชิดหรือการมีเพศสัมพันธ์ของท่านเปลี่ยนแปลงไปเนื่องมาจากท่านรู้สึกง่วงนอนหรืออ่อนล้า					
